# Methods for producing an easily assembled zinc-air battery

**DOI:** 10.1016/j.mex.2020.100973

**Published:** 2020-06-21

**Authors:** Zequan Zhao, Bin Liu, Xiayue Fan, Xiaorui Liu, Jia Ding, Wenbin Hu, Cheng Zhong

**Affiliations:** a Key Laboratory of Advanced Ceramics and Machining Technology (Ministry of Education), Tianjin Key Laboratory of Composite and Functional Materials, School of Materials Science and Engineering, Tianjin University, Tianjin 300072, China; b Joint School of National University of Singapore and Tianjin University, International Campus of Tianjin University, Binhai New City, Fuzhou 350207, China

**Keywords:** Zinc-air battery, Sealing effect, Assembly, air cathodes, Configuration

## Abstract

Zinc-air batteries are considered as the promising alternative to conventional power sources and have received revived research efforts recently due to their high energy density, good safety, environmental friendliness, and potential for low material costs. The design and production of zinc-air batteries is critical to accelerate the commercialization for extending the application range. Herein, we proposed a method for producing plate-type primary zinc-air batteries which apply zinc foil as an example. The proposed method includes the design of an easily assembled zinc-air battery configuration, the preparation of air cathodes and assembly of zinc-air battery. In addition, the galvanostatic discharge performance of the assembled non-flow primary zinc-air battery was tested at a current density of 10 mA cm^–2^. The method can be applied for the production of commercial zinc-air batteries for laboratory research and industrial manufacture for electric vehicles, consumer electronics, and energy storage devices.

• The preparation method for components of zinc-air battery configuration and air cathodes was developed.
• The assembly of the zinc-air battery was proposed.
• Direct evaluation of discharge performance of the zinc-air batteries produced by the method.

## Graphical abstract

**Figure:**
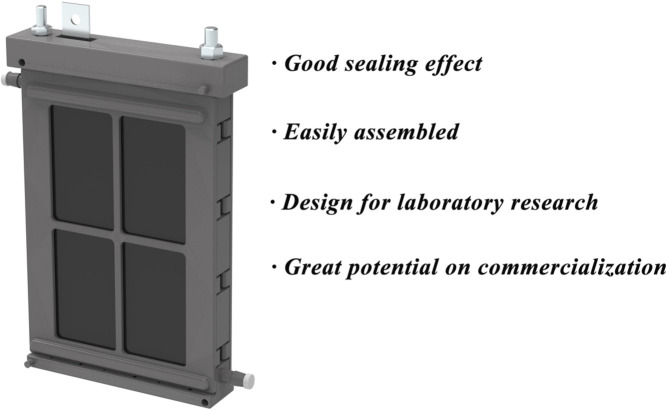

Specifications table

**Table:** 

Subject area:	Chemistry
More specific subject area:	Zinc-air battery
Name and reference of original method:	Production of easily assembled zinc-air batteries with good sealing effect
Resource availability:	Method can be reproduced with regular materials and chemicals used for anodes and cathodes of zinc-air battery construction.

## Method details

Currently, a lot of studies have been devoted to the development of zinc-air batteries [1], [2], [3], and it is highly required to accelerate the commercialization. In this section, details of the method for the production of the proposed zinc-air battery configuration are introduced. Majority of the publications related to research and development of the zinc-air batteries apply the conventional configuration, which consists of multiple plate frames fixed by various bolts located symmetrically [4], [5], [6], [7], [8]. In conventional configuration, adjacent plates generate the clamp force through the tightening of bolts to hold the anodes and cathodes for forming the three-phase reaction interface of the zinc-air battery. However, the conventional configuration is not sufficient for achieving commercialization requirements of zinc-air battery. Since the conventional configuration has low energy density due to the large volume and mass of the package materials, and there is a risk of electrolyte leakage caused by human operation under the uneven clamp force produced by bolts, resulting in complicated maintenance process and inconveniences in the large-scale assembly [9].

Previous studies applied the most common coin-type configuration for producing the state-of-the art, highly optimized zinc-air battery [10]. In addition, some other designs of zinc-air batteries such as, tubular type [11] and plat type [12] have been developed. However, these designs also have some drawbacks for commercialization. For example, although the coin type has been commercialized in the application of hearing-aid, its application range is limited in consumer electronics. The tubular type is only designed for flow batteries which limits its application range, and it is not available for most anode types (i.e., zinc foils). The sealing effect of the plat type is unclear if the liquid electrolyte is applied, and the manual assemble process might be complicated, which reduces the likelihood of mass production.

In our modified configuration, some superiority of proposed zinc-air battery configuration could be reflected when compared with other types. Compared with conventional configuration type, the built-in mechanical fasteners on the battery body are used for replacing the bolts to form a closed internal space with a waterproof silicone ring, which is an easily assembled configuration with good sealing effect for avoiding the risk of the electrolyte leakage. Compared with coin type, the proposed configuration has potential for wider application range such as electric vehicles and energy storage devices in micro grid. Compared with tubular type, the proposed configuration is available for more types of anodes such as zinc foils, zinc sponges and zinc powder anodes. In addition, the proposed configuration can be used for both non-flow and flow batteries. Compared with plat type, the assemble process is easier and could be suitable for mechanical assembly in mass production. The preparation process is listed as follows:

• The design of the components of the battery configuration

**Fig. 1:**
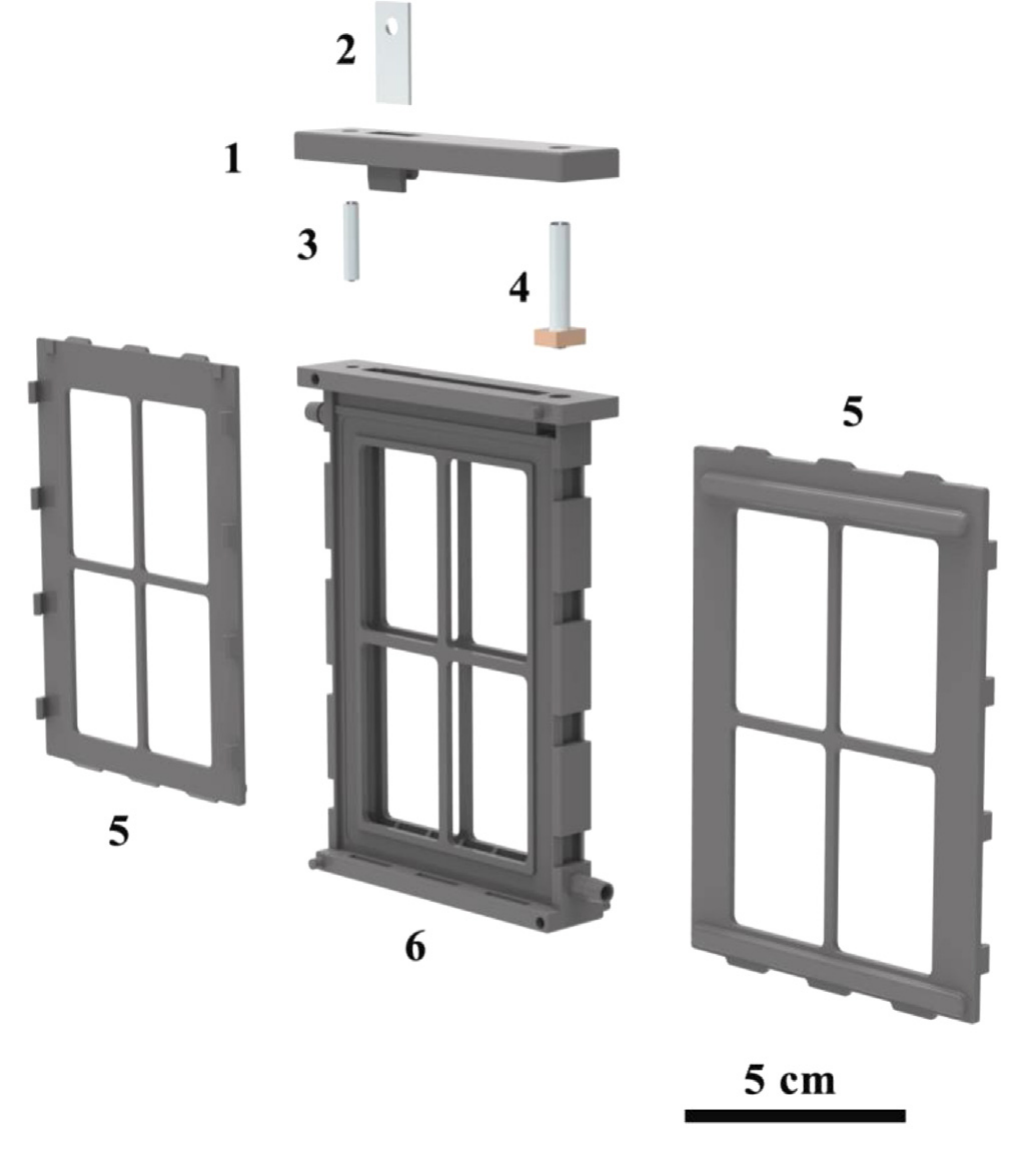
The schematic diagram of components of battery configuration (1 battery cover, 2 metal plate, 3 M4 stud, 4 square-head bolt, 5 pressing plates, 6 battery body).

The editable STEP files of each component of the battery for reference are provided in Supplementary Information, whose dimension parameters can be easily adjusted to meet the specific requirement of practical applications. The battery configuration consists of packaging components and metal accessories. The packaging components of the battery can be produced though 3D printing technology. The STEP files can be downloaded from Supplementary Material, which consists of “PP.STEP” (refers to pressing plates), “BB.STEP” (refers to a battery body) and a “BC.STEP” (refers to the battery cover). In this work, polyamide 12 (PA 12) is chosen for the sealing materials. Previous studies found that PA 12 shows good alkali resistance in 50 wt% KOH solutions [13]. Besides, the packaging components can also be produced through injection moulding for applying other sealing materials (i.e., acrylonitrile butadiene styrene) with good alkali resistance in the mass production. Metal accessories consist of a M4 stud, a square-head bolt made by screwing the M5 stud into a copper block with size of 7.5 × 10 × 3.8 mm, a metal plate with size of 12 × 30 × 1 mm. The schematic diagram of each component of the battery configuration is shown in Fig. 1**.**

• The preparation of the air cathodes

In this section, a convenient preparation method of the air cathodes which can be launched into mass production is introduced. The catalyst on the air electrode is commercial MnO_2_ (α-type, particle size of about 200 nm) purchased from Shanghai Yunfu Nanotechnology Co., Ltd. The catalyst could be replaced by other catalysts according on practice requirements. The air cathodes are prepared though the following steps: (i) The preparation of catalytic layer: MnO_2_ catalyst, XC-72R carbon black and PTFE (60 wt.% emulsion) are dissolved into moderate amounts of ethanol at the weight ratio of 2: 5: 3. The carbon black is applied for enhancing the conductivity of air electrodes, and the catalytic layer could be formed through PTFE binder under this ratio. A slurry of the above mixture is pressured by a roller press to form the catalytic layer. (ii) The preparation of the waterproof diffusion layer: a mixture of XC-72R carbon black, ethanol, and PTFE emulsion with a weight ratio of 4: 4: 2, respectively. The ratio of XC-72R carbon black and PTFE emulsion is consistent with the ref. [14]. A roller press is applied to press the slurry of the above mixture to form the waterproof diffusion layer with the thickness of 0.3 mm. (iii) The current collector (Ni mesh or Cu mesh) is sandwiched between the pressed catalytic layer and a waterproof diffusion layer, and the resulting material is subsequently pressured by a roller press to form the air cathodes with thickness of 0.74 mm ~1 mm. (iv) After drying at 60 °C for 30 min, the air cathodes are cut to plates with uniform size of 112 × 72 mm. The parts of catalytic layer and the waterproof diffusion layer on the top of air cathode plates should be scraped to expose a part of the current collector.

• The assembly of the zinc-air battery with good sealing effect

**Fig. 2:**
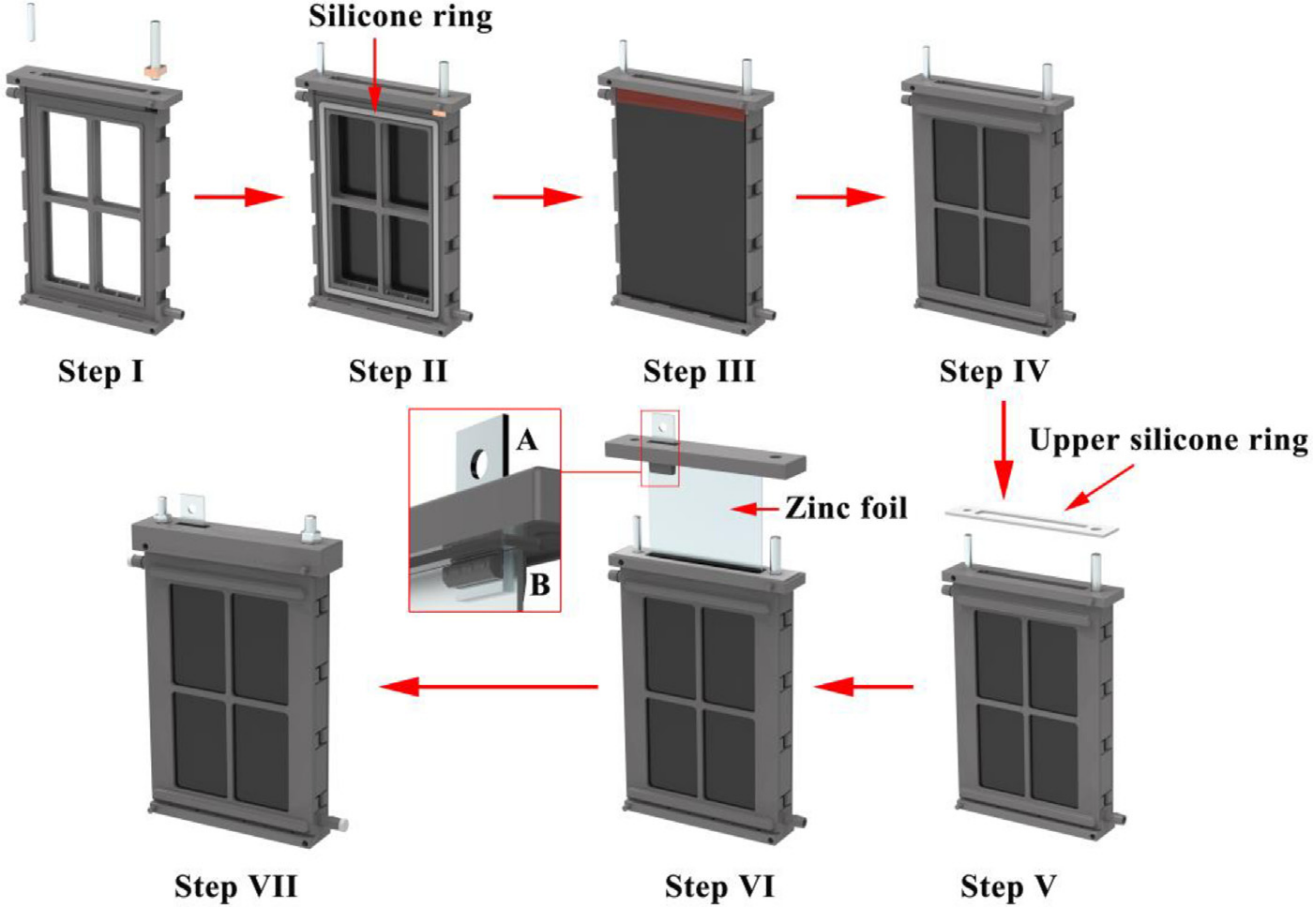
Assembly flow chart of zinc-air battery.

The zinc-air battery is assembled through the following steps: (i) the M4 stud and the square-head bolt are screwed and plugged, respectively, into the corresponding holes on the top of the battery body (Step I in Fig. 2). (ii) The silicone rings (refers to file “SR.STEP”) are placed on the corresponding grooves on either sides of the battery body (Step II in Fig. 2). (iii) The air cathodes are placed on either sides of the battery body above the silicone rings. The exposed part of the current collector on the top of the air cathodes should be contacted with the square-head bolt plugged in the battery body (Step III in Fig. 2). (iv) Pressing plates are pressed on either side of the battery to immobilize the air cathodes via the mechanical fasteners on the battery body to form a closed internal space (Step IV in Fig. 2). (v) An upper silicone ring (refers to “USR.STEP”) is placed on the top of the battery (Step V in Fig. 2). (vi) The metal plate is plugged into the hole of the battery cover, and attaches the zinc anode (zinc foil with size of 60 × 123 × 1 mm) from beneath the battery cover. The above assembly is inserted into the battery body and covers the battery from above (Step VI in Fig. 2). Then, nuts on the bolts are tightened to fix the battery cover. (vii) The electrolyte (which is KOH of 9 mol L^–1^) is injected into the battery though the liquid inlet hole on the battery body. The liquid inlet hole and outlet hole are blocked off though silica gel plugs when the battery is filled with electrolyte (Step VII in Fig. 2).

## Method validation

**Fig. 3:**
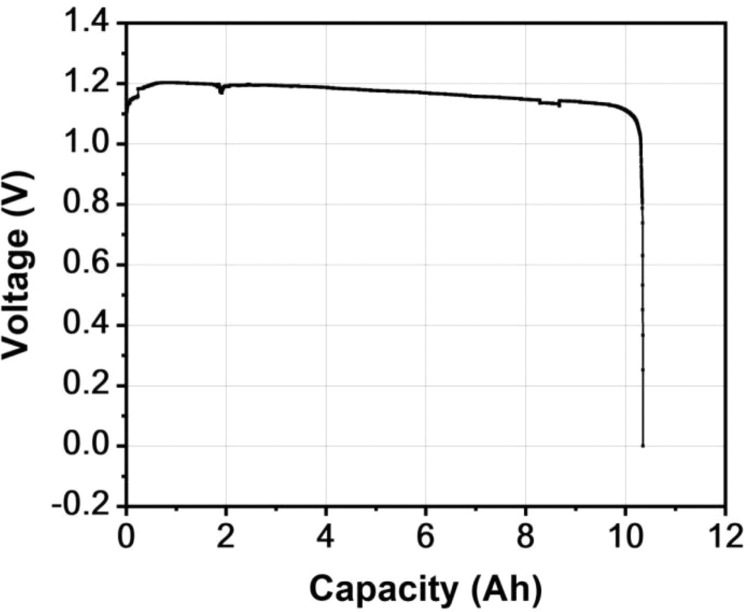
The galvanostatic discharge curve of zinc-air battery at 10 mA cm^–2^.

In order to verify the discharge performance of the zinc-air battery produced by the above assembly method, a galvanostatic discharge test was carried out using a battery testing system (CT2001A, LanHe Instrument Technology Co., Ltd., Wuhan, China) at 10 mA cm^−2^. The test sample applied 9 mol L^–1^ KOH as the electrolyte and the zinc foil with size of 123 × 60 × 1 mm as the anode. The air electrodes of the test sample followed the preparation process of previous section. The galvanostatic discharge curve of zinc-air battery at 10 mA cm^–2^ is shown in Fig. 3. The discharge capacity of the battery is 10.4 Ah. The battery maintains a stable discharge voltage plateau for a long time without electrolyte leakage. In addition, the corrosion and oxide passivation problems on the surface of the current collector in the air electrodes are not obvious when the battery is discharged. The failure of the battery is generally attributed to degradation of the zinc anode rather than the air electrode (including the failure problems of its current collector) because the air electrode usually has a much longer life than the zinc anode [3, 15, 16]. Therefore, it can be concluded the feasibility of the proposed method for producing easily assembled zinc-air batteries with good sealing effect. The catalyst used in the preparation method of air electrodes can also be replaced by some other bifunctional catalysts for producing rechargeable zinc-air battery.

The volume energy density of the test sample is 72 Wh L^–1^, which is normalized by the volume of the whole battery. Latest review article on benchmarking of zinc-based batteries including zinc oxygen batteries or zinc-air batteries summarized the basic characteristics in published articles [17]. However, previously reported energy density of zinc-air batteries was normalized by the mass of anode mixture or consumed zinc anode in most of the studies. Few studies considered the energy density normalized by the volume of the whole battery (including electrodes, electrolyte, and package materials), which is crucial for the actual applications.

It should be noted that the design is also compatible with flowing electrolyte, the flow batteries can be assembled by connecting the battery to the external electrolyte tank through the electrolyte outlet and inlet on the battery body.

## Declaration of Competing Interest

The authors declare that they have no known competing financial interests or personal relationships that could have appeared to influence the work reported in this paper.

## Appendix Supplementary materials

Image, application 1
